# 714 Our Experience with Enzymatic Debriding Agent, Anacaulase, for Burn Injuries: A 14 Patient Case Series

**DOI:** 10.1093/jbcr/irae036.258

**Published:** 2024-04-17

**Authors:** Cole L Bird, Jessica Reynolds, Dhaval Bhavsar

**Affiliations:** University of Kansas School of Medicine, Lees Summit, MO; The University of Kansas Health System, Overland Park, KS; The University of Kansas Health System, Kansas City, KS; University of Kansas School of Medicine, Lees Summit, MO; The University of Kansas Health System, Overland Park, KS; The University of Kansas Health System, Kansas City, KS; University of Kansas School of Medicine, Lees Summit, MO; The University of Kansas Health System, Overland Park, KS; The University of Kansas Health System, Kansas City, KS

## Abstract

**Introduction:**

Background: Enzymatic debridement of burn wounds facilitates early removal of eschar. It has shown to improve healing time and decrease need for surgical intervention. We wanted to review our results of use of an enzymatic debriding agent (EDA - Anacaulase) in deep partial and full thickness burns.

**Methods: Methods:**

We undertook a retrospective review of consecutive 14 adult patients treated with EDA at our Burn Center. These patients received therapy with Anacaulase, a novel enzymatic debriding agent, spanning from the year 2020 to the present day. Burn characteristics, EDA applications details, surgical interventions if any, time of heal, scar assessments were noted. We used Microsoft Excel for a descriptive analysis of the data. We examined continuous variables within specific ranges and provided summaries including median, minimum, maximum values, and percentages.

**Results: Results:**

Patients demographics and burn injury characteristics are included in table 1. We were able to achieve >95% eschar removal with single application in all 14 patients. 57% patients required subsequent skin grafts. The time to achieve 95% wound closure averaged 35.5 days. Scars improved substantially over the study period, as indicated by mean Vancouver Scar Scale scores of 3.8 to 0.5 at 3 and 12 months, respectively. Only 2 patients required scar release surgery due to contracture.

**Conclusions:**

Conclusion: Our review highlights potential benefits of early eschar removal with EDA. 47% patients were able to avoid autologous skin graft closure of deep partial and full thickness burn wounds. EDA (Anacaulase) may add value in care of select patients with deep partial and full thickness burn wounds.

**Applicability of Research to Practice:**

Our review highlights potential benefits of early eschar removal with EDA. EDA (Anacaulase) may add value in care of select patients with deep partial and full thickness burn wounds.

